# Artificial intelligence for antimicrobial resistance: advancing reproducibility, interpretability, and clinical deployment

**DOI:** 10.1093/bib/bbag269

**Published:** 2026-05-31

**Authors:** Sudipta Sardar, Supriya Dash, Puja Roychowdhury, Jaykishan Solanki, Purbita Sikdar, Jayaraman Thangappan, Somenath Dutta

**Affiliations:** Department of Bioinformatics, Pondicherry University, Kalapet, Puducherry 605014, India; Department of Bioinformatics, Pondicherry University, Kalapet, Puducherry 605014, India; Department of Bioinformatics, Pondicherry University, Kalapet, Puducherry 605014, India; Department of Bioinformatics, Pondicherry University, Kalapet, Puducherry 605014, India; Department of Bioinformatics, Pondicherry University, Kalapet, Puducherry 605014, India; Department of Bioinformatics, Pondicherry University, Kalapet, Puducherry 605014, India; Department of Bioinformatics, Pondicherry University, Kalapet, Puducherry 605014, India

**Keywords:** antimicrobial resistance, artificial intelligence, explainable AI, reproducibility crisis, model interpretability

## Abstract

The shift of artificial intelligence for antimicrobial resistance (AI-AMR) from proof-of-concept studies to clinically embedded decision support critically hinges on establishing rigorous reproducibility, interpretability, and evaluation standards aligned with antimicrobial stewardship and patient safety. This review traces the field’s evolution from rule-based gene matching through classical machine learning to deep learning models, foundation models, and generative models and proposes a pragmatic standards framework covering dataset curation, multi-site external validation, transparent model cards, and systematic error-cost analyses. Historically, progress was catalysed by curated resistome ontologies; modern practice demands FAIR-aligned curation, explicit bias and data-leakage audits, and prospective temporal and external geographic validation guided by emerging healthcare AI guidelines. To translate accuracy into safer prescribing, the review advocates cost-sensitive evaluation that quantifies false-positive and false-negative harms, integrates stewardship metrics (time-to-effective therapy, spectrum narrowing, days of therapy), and facilitates continuous post-deployment monitoring. Looking forward, federated learning, multimodal and foundation architectures, generative models for antimicrobial and peptide design, and explainable interfaces usable at the point-of-care are poised to reshape trustworthy and clinically deployable AI-AMR. The review concludes with a checklist for implementers: FAIR and diversity-by-design data curation, prospectively specified multi-site validation, standardised governance-linked model cards, and explicit stewardship-oriented error-cost trade-offs.

## Introduction

Antimicrobial resistance (AMR) poses an escalating global health threat, accounting for approximately 4.71 million deaths in 2021, including 1.14 million directly attributable to resistant infections. Recent forecasts by the Global Research on Antimicrobial Resistance (GRAM) project estimate 39 million deaths from antibiotic-resistant infections cumulatively between 2025 and 2050. Its growing burden extends beyond clinical morbidity and mortality; the WHO projects $1 trillion in additional economic losses by 2050 in the absence of substantial action. Addressing AMR requires approaches capable of integrating complex biological, clinical, and ecological data to inform both surveillance and intervention strategies across interconnected systems, including agriculture, environment, and human health [1].

Artificial intelligence (AI) has emerged as a transformative paradigm in AMR research [2]. The field faces multiple challenges that strongly motivate the adoption of machine learning (ML) and deep learning (DL) approaches: the need to analyse high-dimensional genomic and phenotypic data, deliver rapid and accurate resistance predictions in clinical settings, and overcome the protracted bottlenecks in antimicrobial drug discovery [3, 4].

AI enables the extraction of actionable patterns from large-scale datasets, facilitating resistance mechanism discovery, phenotype prediction, and antibiotic design. Specifically, AI/ML models can analyse large-scale genomic data including whole-genome sequencing (WGS), pangenome data, and metagenomic data to predict bacterial resistance profiles [5]. This capability helps bypass or accelerate traditional, slower phenotypic antimicrobial susceptibility testing (AST). Beyond classification, AI can identify resistance markers and genetic determinants (e.g. mutations, gene presence/absence, structural variants, etc.) associated with AMR, thereby helping to elucidate molecular mechanisms, while predictive ML modelling enables forecasting of emerging resistance patterns, offering early warnings to public health authorities. By training models on historical and current data, AI can predict the spatiotemporal emergence of outbreaks, signalling public health authorities or hospitals to implement targeted interventions (e.g. stewardship, surveillance, and infection control) before widespread resistance develops [6]. AI in this context encompasses a spectrum of methodologies, including classical ML models such as random forest (RF), support vector machine (SVM), and logistic regression (LR); DL architectures like convolutional neural network (CNN) and transformer networks; generative models like diffusion models and generative adversarial networks (GANs); and causal inference frameworks that move beyond correlation toward mechanistic insight.

For AI to have durable translational value in AMR, three foundational priorities must be addressed:

1. Reproducibility, through standardised and transparent pipelines.
2. Interpretability, ensuring clinical and biological intelligibility of predictions.
3. Deployment, embedding AI-driven tools into surveillance networks and healthcare workflows.

Together, these dimensions define a rigorous and responsible path toward leveraging AI to mitigate the global AMR crisis.

Advances in rapid sequencing technologies now enable near-real-time pathogen genomics in clinical settings. Notably, a Guinness World Record for WGS in under 4 h was set in October 2025 by Broad Clinical Labs, exemplifying the feasibility of same-day diagnostics to facilitate precision antimicrobial therapy [7]. These capabilities are underpinned by centralised repositories including the Bacterial and Viral Bioinformatics Resource Centre (BV-BRC), the Comprehensive Antibiotic Resistance Database (CARD), NCBI Pathogen resources, and the recently launched AMR portal [8, 9]. However, their utility is currently constrained by incomplete metadata and heterogeneous curation standards, factors that limit model generalizability and equitable deployment [10].

This review traces the evolution of AI in AMR research, from early alignment-based methods and classical ML to the adoption of deep neural networks and generative models. We explore the integration of diverse data types, including omics and phenotype data, governed by FAIR (findable, accessible, interoperable, and reusable) principles. A standardised repository layout is proposed for reproducible AMR pipelines, alongside best practices for interpretability, model evaluation, and biological validation. Further, we discuss metrics to quantify clinical utility, as well as strategies for deployment, surveillance, and post-deployment monitoring. Selected high-impact case studies illustrate the path forward, concluding with an analysis of current technical challenges and future directions.

## Historical arc: from rule-based to data-driven models

**Figure 1:**
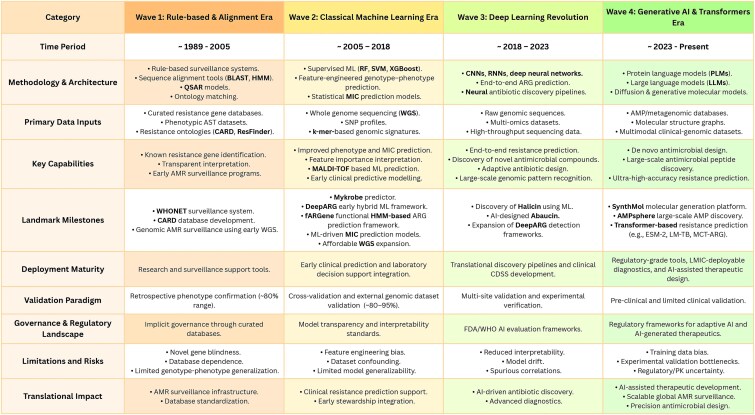
Evolution of artificial intelligence methodologies in antimicrobial resistance research.

Understanding the methodological lineage of AI-AMR clarifies both the capabilities of contemporary models and the origins of ongoing concerns surrounding reproducibility and interpretability. The field has progressed through four broadly sequential methodological waves: (i) rule-based and homology-driven surveillance systems, (ii) classical machine-learning models trained on manually engineered genomic and phenotypic features, (iii) deep-learning and representation-learning approaches capable of modelling complex, high-dimensional resistance determinants, and (iv) transformer-based and generative AI frameworks that enable large-scale resistance prediction, molecular design, and hypothesis generation (Fig. 1). Each transition expanded predictive power and scope, but simultaneously introduced new sources of bias, opacity, and irreproducibility, motivating the need for the technical and reporting standards proposed in this review.

### Rule-based and alignment-based methods

Homology-based detection of canonical resistance determinants constitutes the first standard computational paradigm for AMR, wherein sample sequences are aligned against curated reference collections to identify known genes or mutations. Tools like ResFinder and Resistance Gene Identifier (RGI), supported by the CARD, operationalize this paradigm: a BLAST hit against a designated antibiotic resistance gene (ARG) instantly suggests a plausible mechanism with an interpretable rationale [8, 11]. These methods remain indispensable for surveillance and diagnostic pipelines owing to their speed, transparency, and clinical familiarity.

However, homology searches inherently fail to detect novel genes, sequence variants with altered function, structural rearrangements (e.g. gene truncations or complex plasmid contexts), and regulatory changes that alter expression without sequence identity to known determinants. Furthermore, reliance on curated collections creates a reproducibility challenge: results depend on the precise database version and curation rules used [12].

To extend ARG identification from shotgun metagenomic data, fARGene employs curated, family-specific hidden markov models (HMMs) to probabilistically reconstruct candidate open reading frames (ORFs) from fragmented reads, perform statistical scoring based on HMM alignment likelihoods and conserved sequence motifs, and prioritize novel candidates for phenotypic confirmation via AST in surrogate hosts (e.g. *Escherichia coli*); enabling sensitive detection of both established and divergent ARG variants across complex, uncultured microbial communities [13].

### Classical machine learning methods

To generalize beyond exact matches, researchers employed traditional supervised ML algorithms such as RF, SVM, XGBoost, and LR; mapping engineered genomic features (including k-mers, SNP profiles, one-hot encodings, frequency chaos game representation (FCGR), and SNP presence/absence matrices) to resistance phenotypes and minimum inhibitory concentration (MIC) values, enabling mechanistic interpretation via feature-importance measures. While benchmarking studies highlight their utility, feature engineering imposes scaling limits, as hand-crafted features often miss higher-order sequence contexts, such as plasmid versus chromosomal location, and remain sensitive to variations in assembly/annotation pipelines and sequencing technologies [2, 14, 15]. However, ensemble methods such as XGBoost and RFs generally outperform simpler models in clinical AMR prediction tasks. Beyond resistance prediction, early ML applications also extended toward prospective antibiotic discovery; hierarchical virtual screening frameworks demonstrated that ML could identify novel molecular scaffolds with antimicrobial potential from large chemical libraries laying the conceptual groundwork for modern AI-driven drug discovery pipelines [16].

### Deep learning and representation learning

The development of deep architectures, initially CNNs and recurrent neural networks (RNNs) followed by transformers and large protein/nucleotide language models, enabled end-to-end learning from raw sequences or learned embeddings, enhancing predictive accuracy on large datasets and facilitating transfer learning. Similar deep sequence modelling strategies have also demonstrated strong predictive performance and interpretability in broader RNA bioinformatics tasks [17]. CNNs capture local motifs from one-hot encoded sequences or frequency-based inputs, while transformers and pre-trained language models capture longer-range dependencies and generate embeddings optimised for AMR tasks [18]. Ren *et al*. [5] showed that CNN-based models trained on same encoding outperformed traditional ML models. However, these models exacerbate interpretability concerns: high-capacity networks may learn spurious correlates (lab batch effects, geography, sampling time) and their internal representations are difficult to map to causal biological mechanisms. Consequently, their improved performance underscores the critical need for robust external validation and rigorous reporting of training data provenance and model versions [19]. Recent studies utilizing protein language model (PLM) embeddings highlight both the translational potential and the reproducibility challenges of these architectures. Representative AI-AMR studies spanning classical ML, DL, and rule-based paradigms are summarized in Table 1.

**Table 1:** Representative AI-driven AMR prediction studies across pathogens, data modalities, and validation paradigms.

Pathogen	Antibiotic(s)	Data modality	Model type	Validation strategy	Deployment maturity	Reference
*Nontyphoidal Salmonella*	Tetracycline, ampicillin (multiple classes)	Whole-genome sequencing	Gradient boosted trees (XGBoost)	Internal cross-validation; MIC prediction within ±1 two-fold dilution	Proof-of-concept; ~95% accuracy on NARMS dataset; no prospective deployment	[20]
*Mycobacterium tuberculosis*	Isoniazid, rifampicin (first-line drugs)	SNVs from WGS	Deep denoising autoencoder	Multinational external testing (16 countries); sensitivity 96.3%	Research-stage prototype for multidrug resistance prediction	[21]
*Salmonella* (broiler isolates)	Ciprofloxacin, tetracycline	Gene presence/absence (WGS-derived)	RF	Independent holdout validation; precision 0.91–0.98	Laboratory-validated gene identification; not clinically deployed	[2]
*Escherichia coli*	Multiple β-lactams	Pangenome (core and accessory genes)	RF	Cross-validation; >90% accuracy	Exploratory modelling; adjusted for population structure	[22]
Multiple Gram-negative/positive species (e.g. *E. coli, S. aureus*)	Carbapenems, methicillin, vancomycin	k-mer features from WGS	SCM (rule-based)	Independent test set; >95% accuracy in selected drug-pathogen pairs	Web-based implementation (PATRIC); reference-free genomic prediction	[23]
Multispecies (>7000 genomes)	Carbapenems, methicillin	WGS assemblies	Ensemble classifiers	External holdout validation; >80% accuracy	Surveillance-oriented application; highlights epistatic interactions	[24]

### Generative models and design

Generative modelling—encompassing variational autoencoders (VAEs), GANs, diffusion models, and large language models (LLMs) that constitute the fourth methodological wave in AI-AMR—enables the *de novo* design of therapeutic candidates including antimicrobial peptides (AMPs) and small molecules [25–27]. AMP-Designer exemplifies this potential: the LLM-based foundation framework generated 18 candidate AMPs within 11 days, of which 94.4% (17/18) displayed measurable antibacterial activity *in vitro*, with two candidates further reducing bacterial burden in murine infection models [28]. However, a broader positive-reporting bias exists; models yielding inactive candidates or null in vivo outcomes are rarely published, systematically inflating perceived success rates [29].

Significant translational barriers constrain the clinical utility of generative outputs, as physiological conditions substantially compromise peptide efficacy through protease degradation, salt sensitivity, and assay variability, collectively reducing *in vivo* potency [30]. A principled response embeds stability, toxicity, and robustness objectives directly within model loss functions rather than applying them as post-hoc filters. MOFormer, a conditional transformer for multi-objective AMP design, simultaneously minimizes predicted MIC and haemolysis probability via Pareto-based non-dominated sorting [31]; similarly, Diff-AMP integrates attribute prediction modules within the diffusion pipeline for iterative co-optimisation of activity and toxicity during generation [32]. Reproducibility is further undermined by benchmark bias: Sidorczuk *et al.* [33] showed that the negative data sampling strategy alone confounds performance rankings across AMP prediction models, making cross-study comparisons unreliable. Generalizability is additionally constrained by the near-universal use of susceptible laboratory strains rather than multidrug-resistant clinical isolates in validation assays [34].

Despite more than 5000 AMPs having been identified, only seven FDA-approved AMPs exist, and the vast majority derived from natural sources rather than computational design, highlighting pharmacokinetic limitations, manufacturing challenges and other regulatory barriers [35]. The translational gap remains stark: peptide synthesis generates substantially greater chemical waste per kilogram of product than small-molecule manufacturing; protease stability, haemolytic toxicity, and poor cellular penetration represent liabilities that generative models rarely enforce as hard design constraints [34].

## Data foundations: types, pre-processing, and pitfalls

Robust AI-AMR systems rest on rigorous data stewardship. AMR data are inherently heterogeneous, encompassing genomic reads, assemblies, plasmid structures, phenotypic susceptibility tests, clinical metadata, and multi-omics layers. Each data type entails specific preprocessing requirements and unique failure modes.

### Genomics: raw reads, assemblies, and plasmids

Genomic data encompasses raw sequencing reads, including short reads (e.g. Illumina) or long reads (e.g. Oxford Nanopore, PacBio), draft or finished genome assemblies, annotated gene lists, and variant calls (SNPs, indels, and structural variants) [5]. Read-based classifiers avoid assembly errors and accommodate fragmented samples or metagenomes but require strict control over sequencing errors and mapping artefacts; assembly-based methods facilitate the inspection of gene order critical for determining whether a resistance gene is plasmid-borne yet introduce distinct errors such as misassembled contigs or incorrect genomic placement. Distinguishing plasmid from chromosomal sequence remains a persistent challenge without specialised assemblers or long-read sequencing [35].

Standard feature extraction methods include raw k-mer counts or frequencies, gene presence/absence matrices, single nucleotide polymorphism (SNP) or single nucleotide variant (SNV) features, and language model embeddings generated by LLMs trained on DNA/protein sequences, which may inherit training-data biases, therefore mandating clear documentation of training datasets and model provenance [2]. Notably, sampling bias, particularly overrepresentation of outbreak-associated strains, risks directing models to learn population structure rather than causal resistance mechanisms.

### Phenotypes: MICs, categorical resistance, and AST variability

Phenotypic labels, including MICs or categorical susceptibility calls (susceptible, intermediate, or resistant), are subject to breakpoint discrepancies between CLSI and EUCAST, inter-laboratory variability, and methodological differences (e.g. broth microdilution, automated systems, disk diffusion), generating label noise that substantially affects supervised learning [36]. Isolates near breakpoint thresholds may receive conflicting categorical labels across laboratories or time periods, rendering the ‘ground truth’ unstable. Although incorporating MIC regression models or probabilistic labels can mitigate assay uncertainty, it complicates evaluation. Consequently, explicitly recording the AST method, breakpoint standard, and testing date in metadata should be mandatory [37].

### Other omics, imaging, and multimodal data integration

Proteomic, transcriptomic, and other omics modalities, alongside imaging data, capture resistance mechanisms invisible at the sequence level, including efflux pump induction, antibiotic-induced metabolic reprogramming, and morphological changes under drug pressure. *Visonà* et al. [38] demonstrated an average area under the receiver operating characteristic curve (AUROC) improvement of +0.12 (*P* < .003, Wilcoxon signed-rank test) by combining MALDI-TOF spectra with chemical fingerprints.

However, multimodal integration faces substantial technical challenges beyond simple concatenation. Three key bottlenecks include:

1. Dimensionality and representation mismatch: WGS-derived features encode high-dimensional sparse binary or count vectors (e.g. k-mer frequencies, SNP matrices), whilst MALDI-TOF spectra constitute dense continuous signals requiring baseline subtraction, intensity normalization, and m/z peak alignment, whereas RNA-seq data need variance-stabilizing transformations. These incompatible statistical properties, differences in sparsity, dynamic range, and distributional form necessitate modality-specific encoders with shared latent-space projection [39, 40].
2. Batch effects: Instrument recalibration drift, culture media variability, and inter-laboratory spectral divergence are resistant to standard batch-correction methods (e.g. ComBat) when batch membership is confounded with sample group identity, particularly in MALDI-TOF MS spectra [41].
3. Genotype–phenotype discordance: β-lactamase gene presence correlates with meropenem susceptibility at only 78% sensitivity and 89% specificity, limiting the interpretability of learned cross-modal associations [42].

Incomplete modality coverage in multimodal datasets further necessitates modality-specific encoders with late fusion or cross-attention mechanisms [43]. However, out-of-distribution generalization remains challenging, as species-drug zero-shot settings show noticeable performance drops across all models. These challenges explain why multimodal AI-AMR models frequently fail to generalize beyond controlled studies.

### Public resources: scopes and limits

Centralised repositories such as BV-BRC, CARD, and NCBI RefSeq form the backbone of AI-AMR model development and benchmarking, yet exhibit structural limitations that hinder reproducibility across studies: incomplete metadata, variable assembly and annotation quality, and asynchronous cross-platform database updates, requiring explicit documentation of database versions and curation timelines [8, 44].

More fundamentally, exclusive reliance on curated repositories risks ‘knowledge closure’—the condition in which models become constrained to recognizing only previously catalogued resistance determinants—creates an inherent and widening lag between biological reality and computational annotation. Ancient metagenome analyses have identified novel class B β-lactamases sharing <45% similarity with any known ARG, suggesting that the true resistome extends far beyond current databases [45]. Similarly, evaluation of DeepARG identified 76 novel β-lactamase genes with <40% sequence similarity to existing references, indicating that conventional identity-threshold-based methods can yield substantial false-negative rates when confronted with divergent variants [46]. Integrating unlabelled data through self-supervised pre-training offers a promising strategy to overcome these constraints. Self-supervised PLMs such as PLM-ARG, leveraging ESM-1b trained on ~250 million sequences, overcome these constraints by achieving AUROC improvements of 9.6%–36% and F1-score gains of 40.8%–107.3% over prior approaches [47, 48]. Similarly, DRAMMA predicts resistance genes lacking detectable sequence similarity to known ARGs by incorporating protein properties, genomic context, and evolutionary signals [49, 50]. Moreover, few-shot and fine-tuning strategies enable rapid adoption to emerging resistance mechanisms or newly introduced antibiotics, reducing reliance on exhaustive manual curation and facilitating more responsive model updates.

### FAIR principles, minimal metadata, and typical failure modes

Adherence to FAIR principles is essential for AI-AMR research. The minimal metadata set per record must include sample accession, geographic location, isolation source (e.g. clinical or environmental), AST method and sequencing platform, MIC values and corresponding breakpoint standard, library preparation protocol, assembly software version, and quality control metrics, alongside anonymized patient attributes (e.g. age, comorbidities) and governance statements where applicable. Inconsistencies or the absence of these details leads to mislabelled genotype–phenotype pairs [47, 51]. Four recurrent failure modes must be explicitly avoided: (i) merging datasets with disparate breakpoint standards without alignment, (ii) omitting database version documentation (e.g. CARD, ResFinder), (iii) training on small, localized cohorts that induce overfitting, and (iv) detecting genes using assembly-based methods while ignoring plasmid context.

### Benchmarking splits and shift detection

Ensuring robust evaluation requires carefully stratified validation strategies: internal cross-validation for model selection, temporal splitting (training on historical isolates, testing on recent ones) to simulate drift, and geographic partitioning to assess generalizability across regions [52, 53]. Temporal splitting is most reliable within single-cohort surveillance datasets but carries inherent limitations in aggregated datasets as collection and sequencing timestamps are rarely harmonised across studies. Molecular dissimilarity-based partitioning represents the most realistic paradigm in AI-AMR models. Phylogeny-aware and homology-based partitioning by evolutionary divergence and pairwise sequence identity produces substantially more ecologically valid performance estimates than random splits, directly reflecting deployment scenarios involving previously unseen lineages [54, 55]. SpanSeq operationalizes sequence identity-controlled partitioning at scale to prevent data leakage from shared genomic backgrounds [56]. In AMR databases where dominant clonal lineages are overrepresented, a lineage-held-out split is essential, as models have been shown to degrade substantially when evaluated against novel lineages [55, 57]. For models trained on high-dimensional embeddings, manifold-aware clustering in representation space can further test robustness beyond sequence identity thresholds [58]. While synthetic data augmentation and simulated sequencing errors remain useful for stress testing, they must be clearly distinguished from empirical test sets, and distributional comparisons of k-mer spectra, allele frequencies, and metadata are mandatory prior to deployment. Resources such as the benchmarking collections provided by *Raphenya* et al. [44] facilitate consistent comparisons and offer templates for constructing robust holdout sets.

## The reproducibility crisis in AI-AMR research

‘Reproducibility’—obtaining identical results from identical data, methods, and code—is the cornerstone of scientific validity [49]. In computational biology, this extends to transparent methodologies, openly accessible data, and documented computational environments. Although FAIR principles offer a framework for data stewardship, the field faces a pervasive reproducibility crisis [49, 51].

AI-AMR is no exception, further compounding these challenges through domain-specific complexities. Persistent concerns over model transparency and insufficient external validation are well-documented; notably, a meta-analysis by Tang *et al.* [59] of 25 ML studies reported a pooled area under curve (AUC) of 0.82, yet with substantial heterogeneity (*I*^2^ > 97%), reflecting non-standardised preprocessing. A 2025 systematic review by Ardila *et al.* [60] corroborated these findings, citing recurrent methodological limitations, including retrospective study designs and the absence of randomised controlled trials. Although gradient-boosted decision trees (GBDT) achieved the highest mean AUROC (~0.80), predictive performance remained inconsistent across pathogen–drug combinations.

Domain-specific challenges exacerbated by evolving breakpoint definitions, undocumented preprocessing decisions across WGS pipelines, and reference databases lacking version. Identical isolates may receive disparate classifications depending on analysis date. Annotations may change after reference database update, thus must be explicitly recorded [37, 61].

### Building a reproducible AI-AMR pipeline: principles and practices

**Figure 2:**
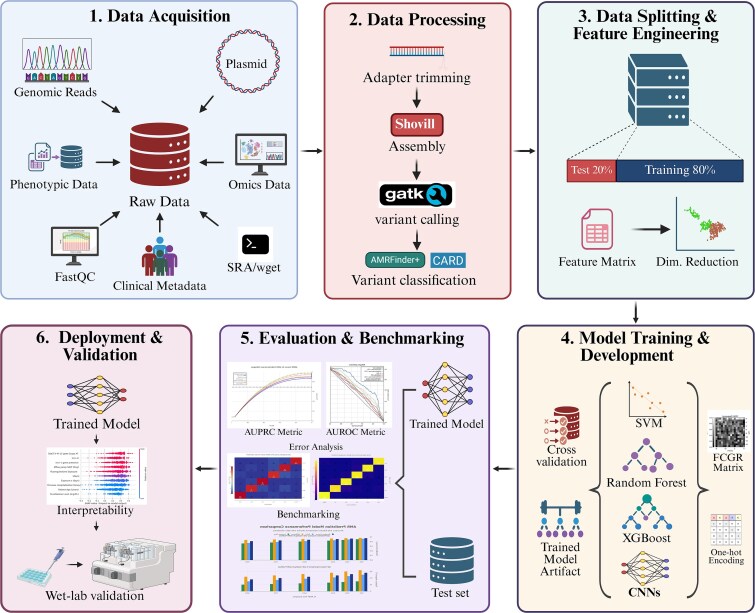
End-to-end reproducible AI-AMR pipeline.

Addressing the reproducibility crisis requires the systematic adoption of technical infrastructure and documentation standards. The following framework integrates established best practices for AI-AMR research and outlines an end-to-end reproducible AI-AMR pipeline spanning data acquisition, standardised processing, model development, benchmarking, and deployment. An overview of the overall reproducible AI-AMR workflow is presented in Fig. 2.

#### Version control, literate programming, and data provenance

The foundation of reproducible research lies in version control. While SVN, BitBucket, and AWS CodeCommit exist, Git remains dominant in bioinformatics due to its performance and integration with popular integrated development environments (IDEs). Version control acts as an immutable ledger, tracking changes to code and scripts to ensure transparency.

Complementing this, literate programming transforms opaque scripts into a comprehensible analytical narrative by integrating human-readable documentation with executable code. Tools like Jupyter notebooks, R Markdown, and Quarto allow researchers to interleave explanatory text with code blocks, capturing not only ‘*what*’ analyses were performed but also ‘*why*’ specific preprocessing or hyperparameters choices were made. Finally, persistent data sharing like Zenodo provides digital object identifiers (DOIs), for deposited datasets, enabling citation and access even in the absence of an associated publication [62].

#### Environment specification and containerization

Containerization technologies are fundamental for reproducible computational biology. Docker provides lightweight virtualization by encapsulating runtime environments and exact software versions [63]. For high-performance computing environments, Singularity (now Apptainer) facilitates rootless container execution, rendering it suitable for shared cluster resources [64]. Collectively, these technologies mitigate environment-dependent discrepancies by guaranteeing identical computational states across diverse infrastructures and timeframes.

Package management via Bioconda provides reproducible dependency resolution for over 8000 bioinformatics packages, encompassing genome assembly, variant calling, and ML frameworks [65]. When combined with strict environment specification files (e.g. environment.yml or pinned requirements.txt), these tools enable the reconstruction of exact computational states years post-analysis. In addition, setting a fixed random seed for pseudo-random number generators (e.g. NumPy or CUDA) is essential to ensure workflow determinism.

#### End-to-end workflow automation

Workflow management systems extend reproducibility to encompass entire analytical pipelines. Tools such as Nextflow enable portable, scalable workflows featuring automatic parallelization and comprehensive provenance tracking [66]. The nf-core initiative exemplifies this approach by maintaining community-curated pipelines that enforce rigorous documentation, testing, and containerization standards [67]. For AMR applications, these systems guarantee the consistency of resistance predictions regardless of the execution environment. Pipelines must encode the complete analytical chain covering quality control, assembly, resistance gene detection, and phenotype prediction, with each step independently containerised via explicit input–output contracts. Notably, the CARD 2023 update implemented standardised 15-character short names specifically to support ML reproducibility [8].

#### Documentation standards and reporting checklists

Model cards provide a standardised framework for reporting ML systems, detailing intended use cases, training data characteristics, evaluation metrics, and ethical considerations [68]. Similarly, data cards extend this rigor to dataset documentation, mandating explicit provenance tracking and the disclosure of known limitations [69]. Consequently, every AMR publication must include comprehensive model cards that document architecture, training data provenance, validation strategies, performance metrics with confidence intervals, and the intended deployment context (Table 2).

**Table 2:** Repository structure for reproducible AI-AMR projects.

Directory	Contents	Format	Purpose
data/	Raw sequences, phenotypes, metadata	FASTQ (SRA), TSV	Complete reanalysis
envs/	Environment specifications	Dockerfile, environment.yml	Reproducible execution
workflow/	Analysis pipeline	Nextflow/Snakemake	One-command reproduction
models/	Trained weights, configs	ONNX, SavedModel	Inference without retraining
docs/	Model card, data card, README	Markdown	Provenance, limitations
results/	Outputs, predictions	TSV with CI	Result verification
tests/	Validation data, expected outputs	pytest	Pipeline integrity

## Interpretability and explainable AI in AMR research

### The interpretability imperative in AMR prediction

The World Health Organization identifies transparency and explainability as foundational principles for AI in healthcare [70]. This transparency is critical in AMR prediction, where algorithmic recommendations dictate antibiotic selection; decisions where delayed appropriate therapy increases septic shock mortality by ~7.6% per hour [71]. Despite the proliferation of AI-AMR studies, a disparity exists between publication volume and methodological rigor: Jung *et al.* [72] reported that only 0.68% (6/882) of healthcare AI articles met quality criteria for explainability, citing inconsistent terminology and a lack of validation as pervasive limitations.

This interpretability deficit is compounded by domain-specific challenges. Clinician adoption hinges on transparency, favouring inherently interpretable ‘white box’ models. However, complex genomic data often necessitates ‘black-box’ architectures. Three specific factors complicate interpretation in AMR. First, models may rely on population structure markers (lineage) rather than causal determinants, failing when resistance emerges in novel genetic backgrounds. Second, resistance often arises from epistatic interactions among multiple genes, and regulatory variants [4]. As Kim *et al.* [2] noted, many ML models treat genes as independent predictors. Third, horizontal gene transfer (HGT) allows rapid resistance dissemination across distant lineages [73], confounding explanations based on stable genotype–phenotype relationships.

### Explainable AI methods applied to AMR prediction

Several Explainable AI (XAI) methodologies have demonstrated utility in AMR applications, ranging from post-hoc feature attribution to inherently interpretable architectures (Table 3), including SHAP-based frameworks, LIME, attention visualization, and rule-based models.

**Table 3:** Explainable AI methods applied in AMR research.

Method	Explanation type	AMR applications	Limitations
Rule-based	Inherently interpretable Boolean conjunctions/disjunctions (1–5 rules)	WGS k-mer-based AMR phenotype prediction (Kover); *Mycobacterium tuberculosis* drug resistance classification (INGOT-DR)	Limited model expressiveness, misses epistatic interactions
LIME	Post-hoc local linear surrogate model	Individual prediction application	Instability, limited global insight
SHAP	Post-hoc global and local feature attribution	WGS ARG identification, MALDI-TOF biomarker discovery	Instability, correlation ≠ causation, high computational cost
Attention Weights	Intrinsic sequence-level attribution	AMP functional motif localization	Attention ≠ importance
Graph rationales	Substructure extraction	Novel antibiotic scaffold discovery against MRSA and vancomycin-resistant enterococci	Search space constraints; limited to molecular graph representation

#### SCM and rule-based methods

The Set Covering Machine (SCM) is an inherently interpretable ML algorithm, used to generate transparent, human-readable predictions. Implemented through the Kover platform, it constructs classifiers as compact conjunctions (logical-AND) or disjunctions (logical-OR) of one to five Boolean rules derived from k-mer features of WGS data, delivering human readable, biologically grounded resistance predictions [74, 75]. INGOT-DR extends this paradigm by formulating rule-based classification as a 0–1 integer linear program inspired by group testing and Boolean compressed sensing [76]. Rule-based methods assume independent feature contributions, however, limiting their capacity to capture the epistatic interactions that account for a significant proportion of resistance phenotypes [77]. They are also prone to capturing population structure artefacts rather than causal resistance determinants and cannot adapt to novel variants without manual curation.

#### LIME

Local Interpretable Model-Agnostic Explanations (LIME) explains black-box models by locally approximating its behaviour with a simpler, interpretable surrogate model such as RF, SVM, etc. [78]. LIME perturbs the input instance, queries the model, and fits a locally weighted linear model to identify which features most influence a given prediction. Despite widespread adaptation in clinical and biomedical AI, very few studies have employed LIME in the AI-AMR domain. Iftikhar *et al*. and Zahra *et al.* applied LIME as an explainable technique for revealing which environmental factors drive ARG abundance. Several methodological limitations constrain the utility of LIME in AI-AMR applications [79, 80]. First, the high dimensionality of AI-AMR models, often encompassing thousands to tens of thousands of features, renders the generation and evaluation of meaningful local perturbations both computationally demanding and susceptible to producing out-of-distribution samples that fail to represent biologically plausible genotypes [81]. Second, LIME assumes feature independence when constructing perturbations, fundamentally problematic for genomic data where genes exhibit epistatic interactions and are subjected to co-selection pressure. Third, repeated applications of the same sample yield different explanations due to the stochastic nature of perturbation sampling process [82], causing instability issues and losing clinical trust.

#### SHAP

Shapley Additive Explanations (SHAP), derived from cooperative game theory, is by far more dominant and the *de facto* standard XAI method in AI-AMR research than LIME, providing both local (one sample) and global (dataset-wide) feature attribution through theoretically grounded marginal contribution estimation [83]. Its applications span clinical electronic health record (EHR)-based resistance prediction (UTI, ICU), genomic/WGS-based ARG identification, MALDI-TOF spectral biomarker discovery, and environmental ARG modelling [84]. Notably, SHAP applied to ML models trained on MALDI-TOF mass spectra from *E. coli, Klebsiella pneumoniae*, and *Staphylococcus aureus* has identified candidate protein biomarkers associated with resistance mechanisms, while *Jain et al.* demonstrated its utility in identifying specific k-mers as resistance drivers in *Pseudomonas aeruginosa* [85, 86]. Though SHAP values for tree models are deterministic, it is not immune to instability—particularly when features are correlated, a prevalent condition in AMR data. While multi-run increases the robustness of SHAP attributions, identifying consistent ‘core’ features and deprioritizing high-variance features, an R package *shapley* was developed applying an alternative approach, which implements performance-weighted mean SHAP values with confidence intervals across entire tuned model grids or stacked ensembles, rather than reporting attributions from a single ‘best’ model. Complementarily, Saadallah [87] proposed a SHAP-guided regularization framework, incorporating two penalty terms: a SHAP entropy penalty encouraging sparse attributions and a SHAP stability penalty ensuring consistent attributions across samples.

#### Attention-based methods and limitations

Attention-based architectures present intrinsic interpretability by identifying sequence positions that contribute most significantly to predictions. Lee *et al.* [88] developed AMP-BERT, demonstrating that attention weights localize to known functional motifs. Similarly, transformer models applied to resistance gene detection highlight sequence regions associated with resistance phenotypes, generating both prediction and mechanistic hypothesis. Recent advances combining attention mechanisms with contrastive learning enable the simultaneous processing of sequence, structure, and annotation features, revealing which information sources drive predictions for specific resistance classes [89].

However, the ‘Attention is not Explanation’ debate demonstrates critical caveats: Jain and Wallace [90] showed that attention weights are frequently uncorrelated with gradient-based feature importance, and that adversarially constructed attention distributions can yield equivalent model predictions - directly challenging attention as a causal explanation. While Wiegreffe and Pinter [91] partially rebutted this claim, arguing that interpretability is condition-dependent rather than categorically absent, and proposing calibration protocols; including variance analysis across random seeds and adversarial attention training to empirically determine when attention may be treated as a reliable signal. But consensus remains that attention weights indicate model focus rather than causal feature importance.

Post-hoc gradient-based attribution methods offer axiomatically rigorous alternatives. Integrated gradients (IGs), computing the path integral of model gradients relative to a defined baseline, satisfy sensitivity and implementation invariance axioms that raw attention weights do not [92]. Tharmakulasingam *et al.* [93] applied this principle in the AMR domain through TransAMR, incorporating a multi-baseline IG pipeline, demonstrating faithful identification of resistance-contributing signatures beyond what attention alone could confirm. Complementarily, as discussed in Section 5.2.3, SHAP has been employed for genome-wide resistance mutation attribution in *Mycobacterium tuberculosis*, where SHAP-derived feature importance independently corroborated motifs identified by attention visualization [94]. For AMP classification, iAMPCN applied SHAP values to validate sequence importance scores derived from convolutional layers, providing orthogonal confirmation of attention-highlighted regions [95]. Enhanced integrated gradients (EIGs) for biology introduced nonlinear paths, group-specific baselines, and statistical significance tests, outperforming DeepSHAP in identifying biologically relevant features [96]. When attention mechanisms are used for interpretation, calibration via IGs ensures models are not merely focusing on sequence-level noise.

#### Explainable graph neural networks

Graph neural networks (GNNs) operating on molecular representations enable antibiotic discovery with integrated explainability. Wong *et al.* [97] developed an explainable, substructure-based approach utilizing Monte Carlo tree search, extracting diagnostic substructures leading to discovery of a novel structural class selective against ‘methicillin-resistant’ *S. aureus* (MRSA) and vancomycin-resistant enterococci, with efficacy demonstrated in murine infection models.

This interpretable approach represents a significant advance over earlier ‘black-box’ discovery methods, such as the identification of halicin by Stokes *et al.* [98] through DL, which, despite demonstrating the model’s generalization capability, lacked a mechanistic explanation, as evidenced by the compound’s low Tanimoto similarity to known antibiotics (~0.21). The transition from black-box to explainable models marks a pivotal maturation toward clinically translatable, AI-driven antibiotic discovery.

### Validation requirements: computation to clinic

XAI predictions necessitate multi-stage validation. Only a few studies complete the full chain of computational, biological, and clinical validation. This validation gap reflects a broader conflation of explanation fidelity with clinical decision utility. Empirically, explanations do not uniformly benefit clinicians: a systematic review of XAI in clinical decision support found that explanations increased trust in only 50% of studies, with some cohorts exhibiting simultaneous increases and decreases contingent on explanation complexity [99]. Cavallaro *et al.* demonstrated that SHAP-supplemented predictions coupled with uncertainty scores reduced antibiotic-phenotype mismatch rates by up to 50% for *E. coli, K. pneumoniae*, and *P. aeruginosa*—one of the few AI-AMR studies coupling XAI with clinician-facing uncertainty quantification [100].

Three clinically consequential risks, however, constrain broader deployment. First, ‘cognitive overload’: high-dimensional SHAP outputs spanning thousands of k-mer or spectral features impose processing demands incompatible with time-pressured clinical workflows; explanations may be skipped entirely or selectively engaged to confirm pre-existing judgment [101]. Second, ‘automation bias’: well-visualized SHAP or attention heatmaps may induce uncritical clinician deference, particularly when explanations reflect population structure rather than causal resistance determinants. Third, ‘instability-induced misinterpretation’: as discussed in Sections 5.2.2–5.2.3, stochastic explanation variance under correlated genomic features is rarely reported in AI-AMR publications, risking clinician treatment of artefactual attributions as definitive mechanistic evidence.

XAI is most defensible when explanations can be prospectively benchmarked against established resistance mechanisms: xAI-MTBDR identified 27 novel resistance-associated mutations across ~40 000 *M. tuberculosis* isolates, several independently corroborated by proximity to drug-target binding sites and broadly concordant with the WHO resistance mutation catalogue, representing a rare computational-to-biological validation chain [94]. Conversely, XAI provides marginal benefit when WGS turnaround precludes real-time use, or when training cohort phylogenetic homogeneity prevents disentanglement of mechanistic from confounded signal. No existing AI-AMR system has completed prospective clinical validation assessing whether explanations measurably improve prescribing accuracy. Closing this gap, through explanation-inclusive usability studies co-designed with infectious disease specialists and antimicrobial stewardship teams, remains the field’s most pressing translational priority.

### Regulatory considerations

Regulatory frameworks increasingly mandate transparency for AI-based medical devices. For instance, the FDA’s guidance on AI/ML-based software as a medical device emphasizes predetermined change control plans and performance monitoring throughout the product lifecycle [102], effectively implying continuous surveillance of prediction accuracy as resistance patterns evolve. Similarly, the European Medicines Agency acknowledges the utility of complex models while prioritizing alternative interpretability approaches when direct explanation proves intractable [103].

Crucially, regulatory acceptance does not demand complete mechanistic transparency; ‘black-box’ models may be permissible when supported by robust validation, rigorous uncertainty quantification, and comprehensive documentation of intended use cases. This emphasis on context-aware validation reflects the consensus that explainability requirements should be proportionate to clinical risk [104]. As XAI methods mature, standardised evaluation frameworks will be indispensable for systematic comparison and regulatory assessment of AI-AMR diagnostics.

## Evaluation: metrics and clinical orientation

### Machine learning metrics

Prior to deployment, it is imperative to evaluate model performance based on both algorithmic robustness and predictive precision. While AUROC and area under the precision-recall curve (AUPRC) are standard metrics for AMR prediction, the prevalence of rare resistant strains creates significant class imbalance, rendering raw accuracy unsuitable. While technically insensitive to prevalence, AUROC can obscure clinical utility—a model may rank resistant isolates reasonably well while exhibiting poor precision for rare resistance mechanisms at clinically actionable thresholds [105]. In AMR prediction, AUPRC is the preferred metric when resistant (positive) class is rare yet clinically critical. However, AUPRC can introduce algorithmic bias by unduly favouring model improvements in subpopulations with more frequent resistant (positive) samples [106]. Matthews correlation coefficient (MCC) emerges as the most robust metric for imbalanced AMR datasets. Chicco and Jurman [107] showed MCC correctly identified poor classifiers (MCC = −0.03) where accuracy (0.90) and F1 (0.95) showed overoptimistic results. For threshold-free evaluation, reporting both AUPRC and AUROC is recommended, with particular attention to prevalence differences when interpreting AUPRC.

### Clinically oriented metrics

Positive predictive value (PPV) and negative predictive value (NPV) are indispensable for guiding clinical decisions, as they are intrinsically linked to the local prevalence of resistance. A high PPV minimizes the risk of unnecessary broad-spectrum treatment, while a high NPV ensures that susceptible predictions do not lead to inappropriate therapy. An RF classifier trained on MALDI-TOF MS spectra achieved PPV and NPV of 0.89 for carbapenem-resistant *K. pneumoniae* and PPV of 0.91 and NPV of 0.93 for colistin-resistant strains across over 8000 clinical isolates [108]. Sensitivity is especially critical for high-risk phenotypes: CarbaDetector achieved 96.3% sensitivity and 86.1% specificity for carbapenemase detection, while an RF model for vancomycin-resistant *Enterococcus faecium* yielded sensitivity and specificity of 0.79 and 0.77 in prospective timewise validation [109]. Turnaround time is equally decisive. While sequence-based models often offer higher accuracy, they incur higher processing time than a MALDI-TOF-integrated ML. Evaluations must therefore explicitly quantify the trade-off between predictive performance and speed [110]. AMR models shall be assessed for their impact on antimicrobial stewardship. A model that marginally reduces uncertainty but materially improves empiric treatment choices may offer greater clinical value than a highly accurate model that fails to alter prescribing behaviour. Therefore, metrics shall be stratified by resistance mechanism and clinical criticality [111].

### Uncertainty quantification: actionable clinical protocols

Models deployed in clinical settings must be well calibrated, with predicted probabilities accurately reflecting observed outcomes. Reliability diagrams and Brier scores quantify calibration, while conformal prediction provides valid uncertainty bounds alongside point estimates [112]. Conformal prediction adds valid uncertainty bounds (e.g. >20% width at 90% coverage flags ~30% AMR cases for wet-lab AST validation or ID specialist referral), boosting trust in high-stakes decisions with Platt scaling preserving discrimination, a critical feature for sepsis prediction models. When models output ‘Uncertain’ predictions, clinical workflows must respond actionably: (i) default to ‘phenotypic AST’ for confirmation when uncertainty exceeds predefined thresholds, (ii) continue to empirical ‘broad-spectrum treatment’ where increased time-to-therapy cause mortality, (iii) ‘refer high-uncertainty’ cases to infectious disease specialists for assessment, and (iv) automatically ‘trigger wet-lab validation’ protocols for research-grade applications.

Reports shall include predicted resistance/susceptibility along with uncertainty measures for easy interpretation; without requiring specialists. Papangelou *et al.* [113] achieved 93.75% empirical coverage at 95% confidence using transductive conformal prediction, identifying 12.5% uncertain cases and detecting 50% model errors. Bayesian neural networks (BNNs) have demonstrated robust resistance prediction with calibrated epistemic uncertainty (AUC 0.92; 47 therapies). Without actionable clinical guidelines, uncertainty quantification has limited practical value [114].

### External validation

External validation is essential for verifying a model’s robustness prior to clinical deployment.

(a) Temporal holdouts: Assess performance on future data from the same facility to simulate drift over time.
(b) Geographic holdouts: Evaluate generalization across disparate hospitals, regions, or countries.
(c) Multi-centre datasets: Capture diversity in patient populations, laboratory workflows, and strain backgrounds.

This testing exposes the model’s brittleness due to genomic drift, evolving resistance mechanisms, or shifts in local epidemiology. A comprehensive validation strategy should encompass multiple external evaluation paradigms, clinical performance metrics, and uncertainty-aware assessments (Table 4).

**Table 4:** Comprehensive metrics framework for AI-AMR model evaluation.

Category	Name of the metric	What it measures	When to use	Strengths	Limitations
Machine learning metrics	Accuracy	Proportion of correct predictions	Only when classes are balanced (rare in AMR)	Simple to interpret	Misleading under severe class imbalance; dominated by the majority class
	AUROC	Global separability between resistant and susceptible isolates	Early exploratory modelling, multi-drug screening, and ranking tasks	Threshold-independent;	
Stable for ranking under imbalance	May hide poor PPV for rare resistance classes				
	AUPRC	Precision and recall for rare positive class	Clinically important resistances; low-prevalence settings	Highlights the model’s ability to detect rare resistance	More variable than AUROC; sensitive to prevalence
	MCC	Overall prediction quality using all four confusion matrix cells (TP, TN, FP, FN); ranges from −1 to +1	Imbalanced datasets; when both resistance and susceptibility errors matter	Reliable under class imbalance; penalizes all error types equally	Threshold-dependent; undefined if a class is absent from predictions
Clinical oriented metrics	Sensitivity	Accurate positive detection for resistance	High-risk resistance (e.g. carbapenemases)	Minimizes false negatives	May reduce specificity
	Specificity	Proportion of truly susceptible isolates correctly identified as susceptible	Inappropriate or over usage of broad-spectrum antibiotics must be minimized	Reduces inappropriate antibiotic treatment	Comes at the cost of sensitivity; may miss resistance calls
	PPV/NPV				
At realistic prevalence	Probability that positive/negative predictions are correct	Clinical deployment in specific hospitals/regions	Reflects real-world utility	Strongly prevalence dependent	
	Time-to-result	Turnaround time for actionable output	Sequencing- versus culture-based comparisons	Directly impacts clinical workflows	May trade speed for accuracy
Calibration and uncertainty	Calibration curve/Brier score	Agreement between predicted probability & actual frequency	Probability-based decision-making	Identifies overconfidence	Requires sufficient data
	Conformal prediction	Valid uncertainty bounds	Settings requiring guaranteed coverage	Provides interpretable uncertainty	May widen intervals under ambiguity
	Bayesian methods	Parameter & prediction uncertainty	Small datasets; prior biological knowledge	Principled quantification	Computationally heavy
Validation strategies	Temporal holdout	Generalization to future isolates	Shifting epidemiology	Captures drift	Needs long-term data
	Geographic holdout	Generalization across sites	Multi-centre deployment	Tests robustness	May require harmonization
	Prospective test set	Real-world evaluation	Clinical deployment trials	Highest validity	Resource intensive
	Wet-lab validation	Experimental verification of predictions	Confident resistance/novel mechanism prediction	Confirms biological relevance	Costly, slow

## Model deployment, surveillance, and monitoring

**Figure 3:**
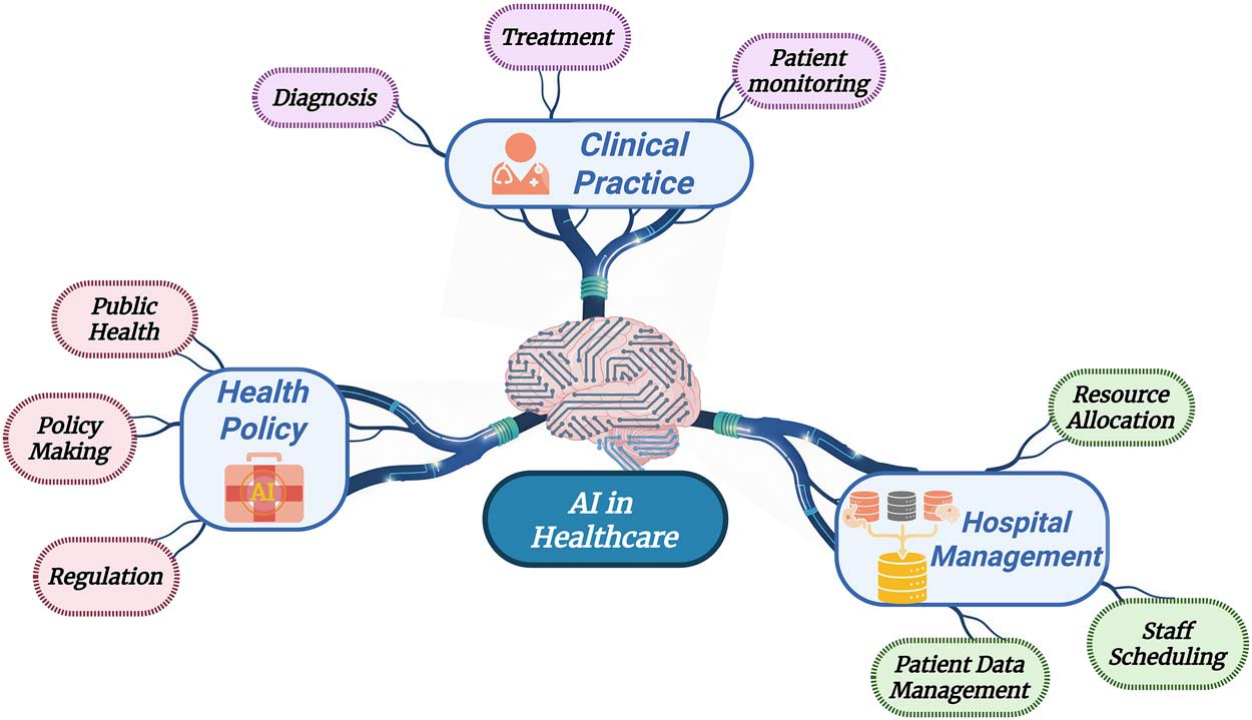
Translational integration of artificial intelligence across healthcare domains.

Translating AI-AMR models from retrospective development into clinical deployment requires coordinated integration across technical, regulatory, and operational domains. AI-driven tools that analyse genomic and microbiological data hold substantial potential to enhance AMR diagnostics, stewardship, and surveillance; however, responsible integration demands rigorous performance monitoring, transparent governance, and sustained human oversight to ensure patient safety and equitable access [115]. The multidimensional integration of AI-driven AMR systems across clinical decision support, public health surveillance, antimicrobial stewardship, and drug discovery is illustrated in Fig. 3.

### From bench to bedside: bridging development and clinical integration

AI-driven clinical decision support systems (AI-CDSS), embedded within EHRs, provide patient-specific guidance on antibiotic selection, dosing, and duration [115]. Weis *et al.* [116] demonstrated that models trained on MALDI-TOF mass spectra achieved higher diagnostic performance and substantially faster results than traditional culture-based methods. The OneChoice® CDSS further exemplifies operational maturity, achieving a 74.59% physician alignment rate and 68.9% implementation rate for treatment modifications in bacteraemia, one of the few AI-AMR systems with documented prescriber uptake [117]. AI-CDSS reduce time-to-effective therapy by automating interpretation of complex diagnostic data and flagging potential outbreaks in real time; however, effective adoption requires interdisciplinary collaboration among clinicians, data scientists, and developers to ensure safety and clinical reliability [118].

**Figure 4:**
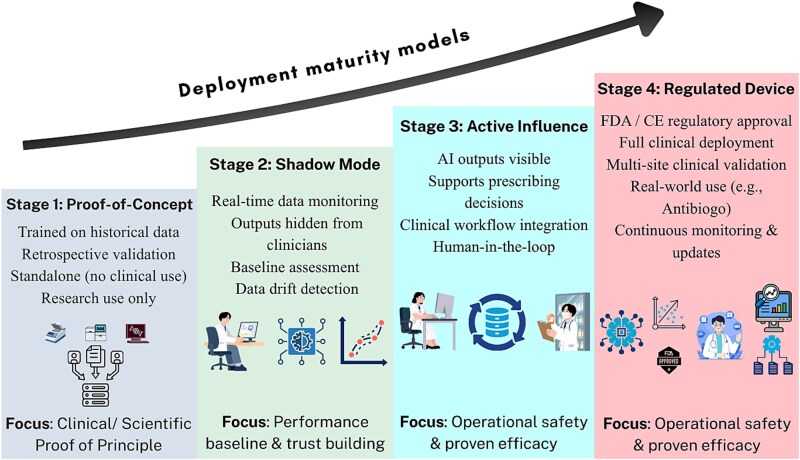
Deployment maturity framework for AI-AMR.

Deployment should follow a structured maturity framework (Fig. 4):

1. Stage 1 (Proof-of-concept): Retrospective validation; constitutes the majority of current AI-AMR publications.
2. Stage 2 (Shadow mode): Real-time performance monitoring against live datasets, with AI outputs withheld from clinicians to establish performance baselines and detect data drift.
3. Stage 3 (Active prescribing influence): AI outputs made visible to clinicians to inform prescribing decisions.
4. Stage 4 (Regulated medical device): FDA-cleared or ce-marked tools in full clinical use, exemplified by Antibiogo, a smartphone-based ML tool for AST zone interpretation, certified in 2022 and deployed by MSF in low-income settings including Yemen and Mali [119].

Successful implementation requires modular microservice architectures (Docker-based containers) connecting AI logic to hospital EHRs via HL7/FHIR interoperability standards, supported by preparation across technical, legal-regulatory, and operational domains.

### CI/CD for surveillance pipelines: ensuring adaptability and reliability

AMR surveillance demands a One Health approach integrating human, animal, and environmental data streams - a paradigm undermined in practice by siloed sectoral workflows, particularly across low and middle-income countries (LMICs) [120–123]. Established international surveillance frameworks include GLASS, CAESAR, EARS-Net, and ReLAVRA, while LMIC-oriented programmes such as SMART (~500 000 isolates from >60 countries over 20 years) and the ATLAS database have substantially expanded geographic coverage. National platforms including Kor-GLASS (South Korea), i-AMRSS (India), and OHE-AMURS (Canada) further illustrate the growing infrastructure for regional genomic surveillance [124–126].

Public health genomic surveillance workflows require reproducible, scalable bioinformatics implementation. Nextflow and nf-core provide containerised pipelines that execute consistently across workstations, HPC clusters, and cloud infrastructure, while specialised platforms such as Pathogenwatch and Microreact automate genomic clustering with geospatial data for outbreak investigation. WGS via Illumina or Nanopore platforms provide a faster alternative to traditional culturing, while integration with ML further improves diagnostic accuracy and resistance trend forecasting. These workflows require standardization before transitioning from research to operational surveillance [127, 128]. Cross-domain applications of AI-AMR are summarized in Table 5.

**Table 5:** Domain-specific applications and impacts of artificial intelligence in AMR research.

Domain	Key findings	References
Clinical practice	Enhances diagnostic accuracy in pathology analysis using MALDI-TOF and genomic sequencing platforms. Improves decision-making in drug discovery and disease management, reduces turnaround time, and strengthens antimicrobial stewardship.	[129–132]
Hospital management	Supports clinical integration of AI-AMR diagnostics into the EHR and LIMS. Automates administrative tasks such as scheduling and billing, reducing errors and staff workload. Improves EHR security and identifies compliance risks through predictive analytics.	[132–135]
Health policy	Enables AMR surveillance at the population level and supports deployment of AI-AMR tools. Identifies ethical risks of implementing AI in healthcare, including data privacy and algorithmic bias. Reshapes policy formulation through data-driven insights and predictive modelling.	[3, 132, 136, 137]

### Federated learning and data governance

Federated learning (FL) offers a principled solution to the tension between data-intensive model training and patient confidentiality, enabling multi-centre collaborative model development by sharing only local model weight updates rather than raw microbiological datasets across institutions. This architecture preserves data sovereignty while allowing models to learn from geographically diverse resistance patterns. Despite this progress, addressing algorithmic fairness and implementing rigorous post-market surveillance remain critical prerequisites for ensuring that AI integration translates into equitable health policy outcomes, particularly where institutional data contributions are skewed toward well-resourced settings [102].

### Operational robustness

Maintaining operational robustness requires proactive management of false-positive and false-negative prediction risks, which directly affect antibiotic selection and patient outcomes. Performance degradation of 0.10–0.25 AUROC over 18 months without retraining underscores the fragility of static models against evolving pathogen populations. Three categories of drift must be distinguished and monitored: model drift arising from pathogen evolution; data drift from changes in laboratory diagnostic methods; and concept drift from the emergence of novel resistance mechanisms with altered genotype–phenotype relationships. Automated monitoring systems must track performance metrics in real time and trigger retraining protocols when accuracy falls below predefined clinical thresholds [138]. Current evidence supports human-in-the-loop (HITL) deployment, particularly during the transition from Stage 2 (shadow mode) to Stage 3 (active prescribing influence), wherein AI provides second-look validation of AST results while a qualified clinician retains final prescribing accountability. Evaluation frameworks must align with stewardship-driven endpoints time-to-effective therapy, spectrum narrowing, and reduced days of therapy rather than relying solely on AUROC, with cost-sensitive metrics explicitly accounting for the asymmetric harms of false susceptibility versus false resistance errors [139].

### Governance and regulatory readiness

Governance frameworks including MI-CLAIM, MI-CLAIM-GEN, TRIPOD+AI, and DECIDE-AI collectively address reporting transparency and lifecycle monitoring, yet none has been specifically adapted for AMR requirements [140–143].

Deployment documentation must explicitly report:

- training data geography and temporal coverage,
- resistance phenotype distribution with breakpoint alignment (EUCAST/CLSI),
- preprocessing pipeline specifications,
- external multi-centre validation outcomes,
- uncertainty quantification methodology,
- drift detection protocols,
- HITL thresholds; and
- prospective validation strategy.

No AI-based AMR tool has yet received FDA clearance, and the existing Predetermined Change Control Plan (PCCP) framework does not accommodate continuously adaptive models that resistance surveillance demands necessitating collaborative development of domain-specific premarket evaluation pathways [144].

## Future directions

**Figure 5:**
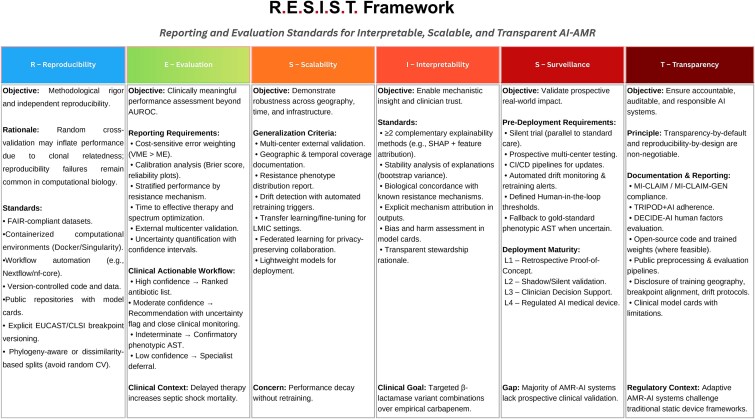
Clinical deployment and monitoring framework for AI-AMR systems.

The convergence of foundation models, autonomous experimentation, and real-time genomic surveillance has positioned AI-AMR research at a translational inflection point; yet systemic gaps persist across the development-to-deployment lifecycle from inflated performance estimates under conventional cross-validation and the high irreproducibility rate in computational biology, through insufficient clinical interpretability and the absence of prospective validation across the vast majority of published studies, to the lack of any FDA-cleared AMR-AI tool and the continued exclusion of low- and middle-income countries from model development [145]. Critically, these deficiencies are not independent: a model trained without biologically grounded partitioning cannot be meaningfully validated prospectively, and a system lacking uncertainty quantification cannot satisfy emerging regulatory frameworks, regardless of retrospective accuracy. The field therefore requires a coordinated transition from addressing individual technical shortcomings toward an integrated evaluation paradigm in which reproducibility, clinical validation, interpretability, scalability, regulatory compliance, and equitable access are treated as co-dependent prerequisites for translational readiness. To this end, we propose the R.E.S.I.S.T. (Reporting and Evaluation Standards for Interpretable, Scalable, and Transparent AMR-AI) framework (Fig. 5), which synthesizes the minimum requirements identified across this review into six interdependent analytical domains: Reproducibility, Evaluation, Scalability, Interpretability, Surveillance, and Transparency. Each domain specifies actionable checklist criteria grounded in quantitative evidence, translational bottlenecks, and governance gaps, providing researchers, reviewers, and regulators with a unified standard for assessing the clinical readiness of AI-driven antimicrobial resistance prediction systems.

### Reproducibility

The next generation of AI-AMR research must embed reproducibility as a structural guarantee rather than a retrospective aspiration. Pre-registration of analytical protocols analogous to clinical trial registration shall become standard practice to prevent *post-hoc* methodological optimization that inflates reported performance. Living benchmark suites, continuously updated with phylogenetically controlled splits and versioned breakpoint annotations (EUCAST/CLSI), will provide the community with standardised evaluation conditions that replace the current fragmented landscape of ad hoc, incomparable assessments. Self-supervised pretraining on unlabelled genomic corpora shall be systematically explored as a mechanism for learning transferable representations that generalize across species, resistance mechanisms, and institutional contexts, reducing dependence on curated labelled datasets whose biases propagate into downstream predictions. Containerised, end-to-end workflow specifications (Docker/Singularity, Nextflow/nf-core) paired with machine-readable model cards must evolve from recommended best practices into enforceable submission requirements for AMR-AI publications.

### Evaluation

Evaluation frameworks must align with stewardship-driven endpoints time-to-effective therapy, spectrum narrowing, and reduced days of therapy rather than relying solely on AUROC or accuracy. The four-filter uncertainty workflow [(1) high-confidence ranked recommendations, (2) uncertainty-triggered phenotypic AST confirmation, (3) continuation of empirical therapy when delay increases mortality risk, and (4) specialist referral or automatic wet-lab validation] should be prospectively validated through randomised controlled trials measuring not only predictive accuracy but also downstream outcomes such as mortality reduction and resistance selection pressure. Cost-sensitive evaluation must account for asymmetric harms of very major errors (false susceptibility) versus major errors (false resistance), especially given evidence that each hour of delayed appropriate therapy in septic shock increases mortality [71]. Future benchmarks should mandate stratified reporting across resistance mechanisms, pathogen species, and specimen types to expose clinically significant blind spots masked by aggregate metrics.

### Scalability

Scalability remains the most consequential barrier separating academic prototypes from deployable infrastructure. Performance degradation of 0.10–0.25 AUROC over 18 months without retraining underscores the fragility of static models, while the persistent *in silico*-to-*in vivo* bottleneck over 5000 catalogued AMPs yet fewer than a dozen approved, with pexiganan, iseganan, and omiganan failing advanced trials demands that future generative architectures embed physiological constraints (serum stability, protease susceptibility, haemolytic toxicity, ionic sensitivity) directly within multi-objective loss functions rather than applying them as *post-hoc* filters. Constructing large-scale paired efficacy datasets measuring activity across serum, whole blood, and pulmonary fluid is equally essential. At the deployment level, foundation models should be distributed in forms enabling lightweight fine-tuning on local epidemiological datasets, supported by federated privacy-preserving architectures and edge-optimised variants accompanied by structured capacity-building that enables meaningful LMIC participation in model governance rather than passive tool adoption.

### Interpretability

The strategic imperative for interpretability extends beyond technical transparency toward mechanistic reasoning that directly enables precision stewardship. Future systems should identify which specific determinant drives a resistance prediction, enabling clinicians to consider β-lactamase inhibitor combinations when a specific enzyme variant is attributed, rather than defaulting to broader-spectrum carbapenems. Complementary explainability methods (SHAP stability analysis, attention-based attribution, graph neural network rationale extraction) should be applied in combination, as single-method explanations risk misleading attributions. Critically, interpretability must be evaluated from the clinician’s perspective: explanations that satisfy computational benchmarks but fail to alter prescribing decisions under time-constrained conditions provide no stewardship value.

### Surveillance

The ultimate translational goal is seamless integration with real-time genomic surveillance: point-of-care sequencing combined with edge-deployed inference enabling same-shift resistance predictions that substantially reduce diagnostic delay. Silent trial models running parallel to standard care without influencing treatment must become the mandatory gateway between retrospective development and active deployment, as they alone detect drift-driven performance degradation invisible to retrospective evaluation. Continuous monitoring pipelines should trigger predefined retraining or alert protocols when confidence degrades below clinically acceptable thresholds. Few-shot and zero-shot learning architectures, leveraging PLMs pretrained on extensive sequence databases, will be essential for detecting emerging resistance variants absent from training sets enabling surveillance systems to anticipate novel mechanisms rather than merely recognizing catalogued ones.

### Transparency

Antimicrobial resistance presents regulatory and governance challenges that differ fundamentally from most clinical AI applications. Unlike static diagnostic models, AMR prediction systems operate within continuously evolving microbial populations shaped by mutation, horizontal gene transfer, antibiotic pressure, and regional prescribing practices. As a result, performance degradation may arise from biological evolution rather than algorithmic instability alone. Governance strategies must therefore account for pathogen ecology, laboratory heterogeneity, and breakpoint reclassification, all of which can alter model validity over time. Conventional oversight models emphasize documentation and initial validation, yet AMR applications require dynamic lifecycle supervision that integrates surveillance data streams, microbiology laboratories, and stewardship programmes. Model evaluation cannot remain a one-time pre-deployment exercise; instead, it must function as a cyclical process aligned with resistance trend monitoring and epidemiological shifts. To enable this transition, governance frameworks in AMR should prioritize version traceability, transparent retraining triggers, cross-institution benchmarking, and shared performance auditing across geographic regions. Without domain-specific oversight architectures that reflect the evolutionary nature of resistance, AI systems risk remaining technically sound but clinically unsustainable. Sustainable clinical integration will depend not only on predictive accuracy, but on adaptive governance capable of evolving alongside microbial resistance itself.

**Key Points**

- Tracing the evolution of AI in AMR research from rule-based homology methods through classical machine learning to deep learning and generative models, this review identifies a pervasive reproducibility crisis evidenced by substantial heterogeneity across published studies, and proposes a comprehensive framework encompassing FAIR-aligned data curation, containerisation, workflow automation via Nextflow/nf-core, and standardised repository structures with explicit model cards.
- This review systematically evaluates XAI methodologies, including SHAP, attention mechanisms, and graph neural networks, across computational, biological, and clinical validation stages, highlighting exemplary validations such as xAI-MTBDR identifying 27 novel resistance-associated mutations across approximately 40 000 *Mycobacterium tuberculosis* isolates corroborated by the WHO mutation catalogue, and graph-based approaches enabling the discovery of a novel antibiotic structural class selective against MRSA and vancomycin-resistant *enterococci* with demonstrated murine efficacy.
- The impact of generative AI on antimicrobial discovery timelines is critically assessed through advances such as AMP-Designer, which generated 18 candidate peptides within 11 days at a 94.4% hit rate compared with traditional years-long discovery cycles, whilst persistent limitations, including restricted *in vivo* validation in susceptible rather than multidrug-resistant isolates, negative-data sampling biases, and pharmacokinetic and regulatory hurdles, collectively necessitate standardised experimental protocols and multi-objective design constraints embedded within model loss functions.
- This review advocates clinically oriented evaluation frameworks extending beyond standard metrics, proposing cost-sensitive approaches that integrate antimicrobial stewardship measures (including time-to-effective therapy and spectrum narrowing), calibration analysis, stratified performance across resistance mechanisms, and explicit harm quantification, informed by clinical evidence that delayed therapy increases septic shock mortality by ~7.6% per hour.
- A deployment checklist is provided addressing microservices architecture, continuous integration/continuous deployment (CI/CD) pipelines, regulatory compliance with FDA and WHO guidelines, automated drift detection documenting AUROC decreases of 0.10–0.25 over 18 months, federated learning for LMIC participation, and human-in-the-loop safeguards ensuring AI complements rather than supplants clinical judgement in antimicrobial selection.

## Data Availability

No new data were generated or analysed in this study. This review synthesizes previously published research, all of which are cited in the references.
